# Automated Disengagement Tracking Within an Intelligent Tutoring System

**DOI:** 10.3389/frai.2020.595627

**Published:** 2021-01-20

**Authors:** Su Chen, Ying Fang, Genghu Shi, John Sabatini, Daphne Greenberg, Jan Frijters, Arthur C. Graesser

**Affiliations:** ^1^ Department of Mathematical Sciences, University of Memphis, Memphis, TN, United States; ^2^ Institute for Intelligent Systems, University of Memphis, Memphis, TN, United States; ^3^ Department of Psychology, University of Memphis, Memphis, TN, United States; ^4^ Department of Learning Sciences, Georgia State University, Atlanta, GA, United States; ^5^ Department of Child and Youth Studies, Brock University, St. Catharines, ON, Canada

**Keywords:** intelligent tutoring system, conversational agents, AutoTutor, mind wandering, disengagement

## Abstract

This paper describes a new automated disengagement tracking system (DTS) that detects learners’ maladaptive behaviors, e.g. mind-wandering and impetuous responding, in an intelligent tutoring system (ITS), called AutoTutor. AutoTutor is a conversation-based intelligent tutoring system designed to help adult literacy learners improve their reading comprehension skills. Learners interact with two computer agents in natural language in 30 lessons focusing on word knowledge, sentence processing, text comprehension, and digital literacy. Each lesson has one to three dozen questions to assess and enhance learning. DTS automatically retrieves and aggregates a learner's response accuracies and time on the first three to five questions in a lesson, as a baseline performance for the lesson when they are presumably engaged, and then detects disengagement by observing if the learner's following performance significantly deviates from the baseline. DTS is computed with an unsupervised learning method and thus does not rely on any self-reports of disengagement. We analyzed the response time and accuracy of 252 adult literacy learners who completed lessons in AutoTutor. Our results show that items that the detector identified as the learner being disengaged had a performance accuracy of 18.5%, in contrast to 71.8% for engaged items. Moreover, the three post-test reading comprehension scores from Woodcock Johnson III, RISE, and RAPID had a significant association with the accuracy of engaged items, but not disengaged items.

## Introduction

Many intelligent tutoring systems (ITSs) implement natural language dialogue and provide one-on-one human-like tutoring in an automated fashion (Woolf, 2010; Graesser et al., 2012; Nye et al., 2014; Graesser, 2016; Johnson and Lester, 2016; Graesser et al., 2017). A well-designed ITS offers personalized and adaptive instruction which is difficult (or sometimes impossible) to implement in a traditional classroom setting with a teacher handling 30 or more students. Some ITSs have been designed to be similar to human tutors in the design of content coverage and tutorial interaction patterns, such as AutoTutor and other systems with conversational agents that have similar architectures to AutoTutor (Nye et al., 2014; Graesser, 2016). Of particular relevance to the present study, ITS designers, human tutors, as well as classroom teachers struggle with how they can best keep the students focused and engaged in content learning. It is well established that engagement is an important component of learning and motivation (Csikszentmihalyi, 1990; D’Mello and Graesser, 2012; Larson and Richards, 1991; Mann and Robinson, 2009; Pekrun et al., 2010; Pekrun and Stephens, 2012). An automated disengagement detector would be of benefit to students, as well as to tutors, teachers, and ITS environments.

Regardless of whether students learn from an ITS, a human tutor, or a teacher in a classroom, students are likely to become disengaged due to various reasons, such as fatigue, environmental distractions, loss of interest, or the stress of falling behind in a course, as will be elaborated below. One strategy that ITS developers have taken has been to increase engagement through gamification (Jackson and McNamara, 2013; Millis et al., 2017), but students can experience disengagement in games just as they do in learning environments without gamification. A different strategy is to detect disengagement as it occurs, so as to better intervene with the student, for example, by redirecting their attention to learning. The prediction and tracking of disengagement can be approached in different ways, such as developing models of disengagement (sometimes operationalized as boredom) from individual difference measures, language, and keystroke analyses (D’Mello and Graesser, 2012; Bixler and D’Mello, 2013; Allen et al., 2016). Tracking students’ disengagement promptly would allow personalized interactions at appropriate times in order to re-engage students. A small number of studies have been conducted with personalized interventions to prevent or interrupt disengaging behaviors and guide an individual learner back on track (Woolf et al., 2010; D’Mello and Graesser, 2012; D’Mello et al., 2012; Lane, 2015; Bosch et al., 2016; Monkaresi et al., 2017). A critical component of such interventions is a built-in disengagement tracking algorithm which can capture behavioral disengagement promptly and accurately.

Disengagement occurs in a number of situations, such as when the student is 1) mind wandering (Feng et al., 2013; Smallwood and Schooler, 2015), 2) distracted by an extraneous goal, 3) impetuously responding in order to finish the task quickly without concern for performance, or 4) “gaming” the learning environment, such as having an adaptive system filling in most of the answers and solutions to problems (Baker et al., 2008). Multiple factors can lead to disengagement or “off-track” behaviors, and these can be voluntary or involuntary. The time-course of completing a task is also an important consideration. For example, students might begin a learning session in an ITS with some level of interest and enthusiasm, but boredom or fatigue may creep in as the session progresses, as the novelty of the system fades, or when they have difficulty comprehending as the material becomes progressively more complex. The latter is of particularly relevance to this study, as disengagement is negatively related to reading comprehension (Millis et al., 2017).

Disengagement also presents a problem for researchers interested in evaluating learning and performance. Time on task alone (e.g., time spent on one question, problem, text, or session) can be considered contaminated by disengagement in contrast to diligent efforts to complete the task. Disengaged students may take too long a time (thinking about something irrelevant to the reading task) or too short a time (quickly finishing the question or session without comprehension) on a given question, problem, text or session. That is, a disengaged reader can be extremely slow or fast in processing during a learning task with low performance. Data analyses that do not consider the abnormal reading time due to disengagement may lead to unreliable or even misleading results. Moreover, a simple unidimensional measure of time is not sufficiently diagnostic of disengagement because both very fast times and very slow times can be signals of disengagement.

Existing disengagement/engagement detection methods that focus on mind wandering have applied supervised learning approaches to train models using self-reported mind-wandering (Mills and D’Mello, 2015; Millis et al., 2017; Bosch and Dmello, 2019) or use of commercial eye-tracker to automatically detect mind-wandering (D’Mello et al., 2012; Hutt et al., 2019). Another approach uses researcher-defined disengagement when examining student performance profiles over days or weeks, such as a student who is inactive for at least seven consecutive days (Chen and Kizilcec, 2020). In the self-reported approach, the participants are probed during reading with a stimulus signal, upon which they report whether or not they are mind-wandering. Self-reported mind-wandering is not considered a practical tracking system for detecting concurrent disengagement, however, because such self-reports could interfere with the learning process. Moreover, these self-reports may have a response bias to the extent that disengaged students may not admit that they have been disengaged due to social desirability bias (Holden and Passey, 2010). An alternative to self-report was proposed by Beck (2005). In this approach, item response theory was used to predict the probability of a correct response based on the response time and then estimate the probability of disengagement given the probability of being correct for engaged vs. disengaged students. However, Beck's method requires a large sample size to build a model that accounts for inter-student and question variability since a large number of parameters were introduced. This method is therefore also not suitable for tracking disengagement during tutoring since the sample size required is only attained after a student completes a large number of questions. Additionally, existing methods mainly focus on detecting students that are disengaged rather than a specific period where a student gets disengaged (Bulathwela et al., 2020). It would be more helpful if we can detect the time period where students start to get disengaged and re-engage them promptly.

Graesser, Geenberg, Frijters, and Talwar (submitted) identified questions that a student answers that are within the student's “zone of engagement”. These “engaged question-answer observations” included questions that were answered neither too slowly nor too quickly (within ± 0.5 standard deviation of mean log of response time), based on a student's personal average speed of answering questions in a lesson. The participants were struggling adult readers (*N* = 52) who completed up to 30 lessons in a computerized learning environment (AutoTutor) that was part of a 4-month intervention that trained them on comprehension strategies. Answer time alone was not sufficient to identify the incidence of disengagement because accuracy in answering the questions is obviously important. Therefore, questions outside of the zone of engagement, “disengaged question-answer observations” were defined as being answered incorrectly and too quickly or slowly. This approach to identifying disengaged observations was completed after the 4-month study was completed. Unfortunately, this method, however, is not suitable for monitoring concurrent disengagement since disengaged question-answer observations can only be detected at the end of a reasonably large sample of lessons. That does not allow an intelligent learning environment to give feedback and guidance to the learner when disengagement is detected. Moreover, if a question is incorrectly and slowly answered, it may not necessarily indicate disengagement. It is possible that a student is at the very early stage of learning new material and spending time in productive comprehension activities. Nevertheless, an approach to detecting disengagement based on the accuracy and time to answer questions during training is a reasonable approach to building a disengagement tracking system. It does not require special physiological or neuropsychological sensing devices, eye tracking, self-reports of engagement, or machine learning with supervised training that cannot scale up to real-world applications. The approach would be more useful to the extent it could detect disengagement in a smaller time span, such as a minute or two.

In this paper, we propose an unsupervised self-learning algorithm to monitor whether a student is engaged in answering questions within lessons of a conversation-based intelligent tutoring system. The system is *AutoTutor for Adult Reading Comprehension* (AutoTutor-ARC), a version of AutoTutor to teach adult learners reading comprehension strategies. In AutoTutor systems, a tutor agent and optionally a peer agent hold conversations with a human student. When the conversation has two agents (tutor and peer), the conversations are called *trialogues*, as opposed to tutor-student dialogues (Millis et al., 2011; Graesser et al., 2014). Similar three way interactions between two agents and humans have been designed in other learning and assessment environments (Danielle et al., 2006; Jackson and McNamara, 2013; Zapata-Rivera et al., 2015; Lippert et al., 2020) and even in museums (Swartout et al., 2010). Disengagement is detected in an algorithm that considers the time that an adult student spends answering a question, and his/her performance accuracy (i.e. whether a question was answered correctly). Disengaged learners tend to spend too long or too short a time on a particular question and perform poorly on the question or adjacent questions (Greenberg et al.; Millis et al., 2017).

The proposed algorithm starts out by considering the first three to five correctly answered questions to estimate the students’ engagement pace within a specific lesson. The underlying assumption is that students are engaged at the beginning phase of a lesson and most likely performing well. Engagement time to answer a question can be estimated at this early phase of a lesson and serve as a standard of engagement for a particular student on a particular lesson. Based on the standard, the algorithm subsequently tracks students’ performance to identify questions for which they exhibit disengagement by virtue of being inaccurate or too fast or slow compared with the engagement pace. The underlying assumption is that students are engaged at the beginning phase of a lesson but periodically become disengaged in latter phases when they are bored, confused with difficult material (e.g. sometimes due to the increment in levels of difficulty designed in AutoTutor), or mind wandering. We implemented the proposed algorithm to predict/monitor disengagement in AutoTutor-ARC. Our results show that items that the detector identified as the learner being disengaged had a performance accuracy of 18.5%, in contrast to 71.8% for engaged items. Moreover, three post-test reading comprehension scores from Woodcock Johnson III, RISE, and RAPID had a significant association with the accuracy of engaged items, but not disengaged items. The development of DTS algorithm is motivated by response time and performance data generated by the users of AutoTutor-ARC system. DTS has not been used in any intelligent system yet. The validation analyses in the manuscript can be considered as a “low stakes” application of DTS. If successful at detecting disengagement, the proposed real-time disengagement tracking system could be of value in enhancing learning efficiency in future AutoTutor-ARC systems, if it can be coupled with interventions during a lesson that re-engage a disengaged student. The algorithm could also be applied to other computer-based learning or assessments that utilize a question-answer environment.

## Data

### Description of AutoTutor-ARC

There are many versions of AutoTutor on various topics, strategies and skills that help students learn by holding a conversation in natural language with computer agents (Nye et al., 2014; Graesser, 2016). AutoTutor-ARC was developed to help adult learners improve reading comprehension. It was first implemented as part of an intervention study conducted by the Center for the Study of Adult Literacy (CSAL, http://csal.gsu.edu). AutoTutor-ARC is a web-based intelligent tutoring system with 30 lessons focusing on building reading comprehension strategies (Graesser et al., 2016b). In each lesson, the learner engages in tutored instruction on comprehension strategies by having trialogue conversations with two computer agents (a tutor and peer). Through the three-way conversations, the learners are provided not only with instructions on reading comprehension strategies, but also guided and hopefully motivated by the computer agents during the learning process.

The lessons typically start with a 2–3 min video that reviews the comprehension strategy that is the target of the lesson. After the review, the computer agents scaffold students through the learning by asking questions, providing short feedback, explaining how the answers are right or wrong, and filling in information gaps. Since adult learners in AutoTutor typically have substantial challenges in writing, AutoTutor tends to rely on point-and-click (or touch) interactions, multiple-choice questions, drag-and-drop functions, and other conventional input channels. The learner chooses the answer by selecting an answer, while the peer agent sometimes gives his answer by talking. Flow within each lesson is driven by either a fixed sequence or contingent branching. The first set of question-answer items within a particular lesson is the same for all students who take the lesson. Fixed sequence lessons deliver the same set of conversational questions to all students, independent of their performance throughout the entire lesson. Contingent branching lessons start out with questions and materials at a medium level of difficulty, but subsequently shift to harder or easier materials/questions depending on their performance on the medium difficulty material (Graesser et al., 2017). For example, 11 of the lessons have multi-sentence texts. For each of these multi-sentence texts, students read the text and then are asked approximately 10 agent-based conversational questions in a fixed sequence for the text. If a student performs well on the 10 questions, then the student receives a second more difficult text with a fixed sequence of approximately 10 questions; students below mastery threshold receive a relatively easier text with approximately 10 questions. These questions are consecutively ordered within one lesson that a student receives. For example, if the first text has 10 questions, coded 1 to 10, and then the second text's questions start with 11 and go to 20. Thus, a lesson may contain questions of two different difficulty levels, e.g. “medium and easy” or “medium and hard”. Some lessons have contingent branching but there is a smaller span of text to be comprehended, such as the comprehension of sentences or words in a sentence. Again, these question items start out medium but branch to more easy or difficult items depending on the student's performance. Accuracy (correct/incorrect) and time spent (called *response time* (RT) later in the manuscript) on each question is recorded per lesson per student.

### Participants and Design

The data sets used to test the proposed algorithm were taken from three waves of an intervention study in two medium sized cities. Participants were 252 adult learners who were offered approximately 100 h of instructional intervention designed to improve their reading skills. The intervention period lasted over 4 months and was implemented in hybrid classes, which consisted of teacher-led sessions and the computer-based AutoTutor-ARC sessions. Their ages ranged from 16 to 74 years (M = 42.4, SD = 13.9) and 74.6% were female. The reading level of participants ranged from 3.0 to 7.9 grade equivalencies. On average, the 252 participants completed 30 lessons. The adult students were also assessed with three standardized tests of comprehension before and after the instruction.

The AutoTutor-ARC content (i.e., lessons and texts) were scaled according to Graesser and McNamara's (2011) multilevel theoretical framework of comprehension. The framework specifies six theoretical levels: word (W), syntax (Syn), the explicit textbase (TB), the referential situation model (RSM), the genre/rhetorical structure (RS), and the pragmatic communication. Words and syntax represent lower level basic reading components that include morphology, word decoding, word order and vocabulary (Rayner et al., 2001; Perfetti, 2007). The TB level focuses on the meaning of explicit ideas in the text, but not necessarily the precise wording and syntax. The RSM level refers to the subject matter and requires inferences to be made on the explicit text and it differs by text type. For example, in narrative text, the RSM includes the characters, objects, settings, events and other details of the story; while in informational text, the model corresponds to substantive subject matter such as topics and domain knowledge. Rhetorical structure/discourse genre (RS) focus on the differentiated functional organization of paragraphs and type of discourse, such as narration, exposition, persuasion and description. Among the four theoretical levels, TB, RSM and RS are assumed to be more advanced and difficult to master compared to words and syntax (Perfetti, 2007; Cain, 2010)). AutoTutor taps all of these levels except for syntax and pragmatic communication. Each lesson was assigned a measure of the relevance to one to three of the four theoretical levels according to the extent to which the level was targeted in this lesson. The assigned codes were primary, secondary, tertiary or no relevance of a component to a lesson, corresponding to a relevance score of 1.00, 0.67, 0.33 and 0.00 respectively (Shi et al., 2018). In this study, we simply consider the primary theoretical level that characterizes the lesson. Table 1 specifies the primary theoretical levels that characterize the 34 lessons (Actually 34 lessons were designed in CSAL, but only 30 (or less) lessons were assigned to the 252 learners in pilot studies).

**TABLE 1:** Distribution of Primary Theoretical Levels Across the 34 lessons.

Theoretical level	Number of lessons	Lesson names
Word (W)^a^	4	4-Word Parts, 6-Word Meaning Clues, 7-Learning
		New Words, 8-Multiple Meaning Words
Textbase (TB)	4	9-Pronouns, 12-Key Information, 16-Main Ideas,
		17-Persuasive Texts
Referential Situation Model (RSM)	15	1-Text Signals, 10-Non-Literal Language, 11-Review 1,
		13-A Personal Story,14-Connecting Ideas, 15-Story
		Maps,18-Review 2, 27-Complex Stories,28-Inferences
		from Texts, 29-Complex Persuasive Texts,
		30-Forms and Documents, 31-Job Applications,
		32-Searching the Web, 33-Using Email, 34-Social Media
Rhetorical Structure (RS)	11	2-Purpose of Texts, 3-Complex Texts, 5-Punctuation,
		19-Claims vs. Support, 20-Problems and Solutions,
		21-Cause and Effect, 22-Describing Things,
		23-Compare and Contrast, 24-Time and Order,
		25-Steps in Procedures, 26-Review 3

^a^Syntax is grouped into the words (W) category in Table 1.

## Methodology

### Algorithm of Disengagement Tracking System

**FIGURE 1:**
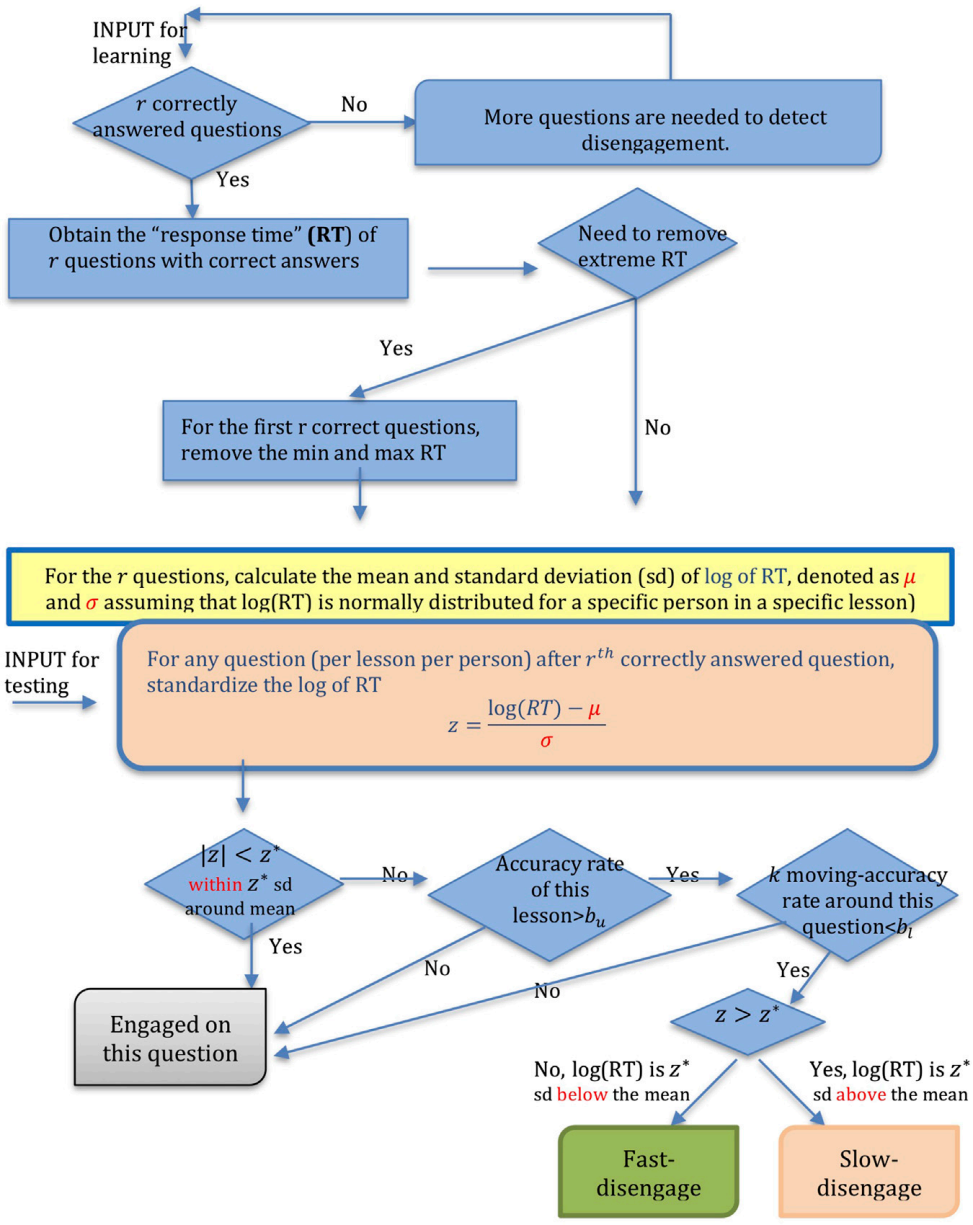
Algorithm Flow Chart of Disengagement Tracing System.

An automated disengagement tracking system (DTS) is ideally personalized to the response times of individual students who work on a particular lesson. For any given student, a DTS should adapt to the learner's pattern of engaged performance, that is, the typical response time when engaged in attending to lesson content. Disengagement is detected when a student's performance (reading time or accuracy in answering questions) significantly deviates from this ‘typical’ pattern. In AutoTutor-ARC, a student is asked to read a text or sentence and to answer questions that are woven into the conversation between the two agents and the student. The system records the time that this student spends on each question and whether a question is answered correctly (1: correct, 0: incorrect). The amount of time a student takes to respond to a question, namely the response time (RT), is one behavior that can be used to determine whether a student is disengaged while working on this question. Performance suffers when the student is disengaged. One indication that students are disengaged is that they respond too fast or too slow (relative to their personalized typical RT) on a question. Too short or long RT does not necessarily mean “disengagement”, since other factors influence RT. However, the short and long times can often be signals that probabilistically predict disengagement. For example, a short RT could be impetuous responding or gaming the system, whereas a long RT may be a difficulty level shift in texts/questions, mind wandering, or a personal bio break. To supplement the validity of RT indicator, we can consider another indication of disengagement: a significant drop in the correctness rate of a student. Thus, the DTS detects questions that a student is disengaged using both indications together. A flow chart of the DTS algorithm is shown in Figure 1.

The top half of Figure 1 demonstrates the process of identifying parameters of response time distribution of engaged question-answer observations (i.e. learning stage in Figure 1), while the bottom half provides a logical procedure for disengagement detection using the parameters estimated in the learning stage (i.e. detection stage in Figure 1). Evidence shows that engagement wanes as time passes and disengagement usually occurs in the later phase when a subject withdraws from the commitment to task goals (Millis et al., 2011; Hockey, 2013). Hence, it is reasonable to make an assumption that a learner is more likely to be engaged at the beginning of a lesson. DTS learns a student's engaged RT from the first few questions within a lesson and uses it to identify questions with abnormal (or disengaged) RT later on.

In the first phase at the beginning of a lesson, the DTS obtains the distribution of a student's engaged RT on questions. Response time is usually right-skewed, as is the current data set, so a log transformation was applied to make the data resemble a normal distribution. We assume an engaged student's log(RT) on a question within a specific lesson is normally distributed with mean μ and standard deviation σ. In practice, most per person and per lesson log(RT) distributions meet the normality assumption, or very close to it. This assumption was checked and validated before we started our analysis. To this end, we make two assumptions: 1) students tend to be engaged at the beginning of a lesson when answering the first few questions, and 2) if a student correctly answered a question, he/she is likely to be engaged. It is possible that students may be disengaged at the beginning of a lesson due to a variety of reasons. Alternatively, students may correctly answer a question by chance when they are actually disengaged. However, there is a low probability that a learner is disengaged and correctly answers several questions by guessing or randomly clicking. The proposed method focuses on the first few (e.g., five) questions that were correctly answered and assumes that the students were engaged while working on these questions that were correctly answered. Even though there might be very few questions that were mistakenly counted as “engaged” (when they should be counted as “disengaged”), the results of the proposed method should not be substantially affected since we excluded the extreme (minimum and maximum) RT of the initial questions, as will be elaborated below.

We will now turn to some of the mathematical specification of the DTS algorithm. Suppose that students are engaged on the first *b* correctly answered questions and start to get disengaged at the $s^{th}$ question some time point later. Presumably, *s* should be greater than or equal to *b* for the system to learn a user's engaged response time in a specific lesson. If s≤b, the algorithm will specify that you will need more questions to detect disengagement. If s>b, DTS will automatically treat the response time of the first *b* correctly answered questions as engaged response time. If there are less than *b* correct question-answer observations up to the $s^{th}$ question, we tentatively use question #2 to question #*b*'s response time as engaged response time instead. We excluded question #1 since the users usually take extra time to read the text in the first question and spend much longer time than usual. Let *I* be the first *b* correctly answered questions, whereas μ and σ are estimated by

$$\hat{\mu }=\frac{\sum_{i\in I}log\left(RT_{i}\right)}{b}$$

and

$$\hat{\sigma }=\sqrt{\frac{\sum_{i\in I}{\left(log\left(RT_{i}\right)-\hat{\mu }\right)}^{2}}{b-1}}$$

respectively. As we know that sample mean and standard deviation is very sensitive to outliers, the algorithm provides an option to data analysts whether they would like to remove the minimum and maximum RT among the first *b* correctly answered questions if they believe that there are extreme outliers in the log(RT). A student is potentially disengaged at question *s* if the standardized response time satisfies

$$|z|=|\frac{log\left(RT_{s}\right)-\hat{\mu }}{\hat{\sigma }}|>z^{\text{*}},$$

where $RT_{s}$ is the response time at the $s^{th}$ question and $z^{\text{*}}$ represents the number of standard deviations that the candidate log(RT) departs from the engaged mean of log(RT) to be considered as potential disengagement. A student is slow-disengaged on a question if $z>z^{\text{*}}$, and fast-disengaged if $z<-z^{\text{*}}$. It is known that for normal distribution, 95% should fall within 2 standard deviations and 99.7% should fall within 3 standard deviations. Thus, if we set $z^{\text{*}}=3$, the probability that a student is falsely tested to be disengaged is only P(|z|>3)=0.03% given the student is actually engaged. Data analysts are free to choose the value of $z^{\text{*}}$ that are appropriate for their study. The choice of $z^{\text{*}}$ should be guided by users’ tolerance of false positives. Theoretically, the probability of false positives (i.e. false disengagement) would be 5% if $z^{\text{*}}=2$. In our analysis, we chose r=5 and $z^{\text{*}}=3$. Specifically, we computed the mean and standard deviation of the log(RT) of the first five correctly answered questions. It is possible that one question might be answered correctly by accident. To take this into consideration, we dropped the highest (and lowest) reading time before calculating the benchmark statistics. In the current dataset, for the five correctly answered questions, we removed questions with the highest (and lowest) response time and calculated the engaged mean and standard deviation of log(RT) with the remaining three questions. If the student has less than 3 (correctly answered) questions, the system will use the response time of question 2 to 4. In our analysis with AutoTutor-ARC data, a student is suspected to be disengaged on a question (too fast or too slow) if the log of response time on this question is below or above 3 standard deviations from the engaged log(RT).

To have an adaptive DTS, the mean and standard deviation of engaged RT should be personalized for different students in different lessons. This means that 20 s may be an engaged RT for student *X*, but may be too fast to be engaged for student Y on the same question. Learners usually get disengaged for a variety of reasons. They may even get disengaged at different questions in different trials of the same lesson. Furthermore, an individual's reading ability may vary depending on the characteristics of the texts (e.g. difficulty, type) included in each lesson. Because of these sources of variation, the system is required to learn engaged RTs (or reference behaviors) for each learner within each lesson.

Disengagement detection that is only based on response time would lead to a large number of false positives. Some lessons have a small number of “confidence-boosting” questions, which means learners will respond more quickly with high accuracy to these questions than to others. Students may slow down in subsequent questions that are more challenging, which may be falsely detected as disengagement by an DTS that only relies on response time. Other than “abnormal” response time, another important signal of disengagement is that disengaged students usually perform poorly since they are not focusing on the question. If a good student (whose overall performance within the lesson or up to the current question is high, e.g. greater than 80%) responds to a particular question too fast or slow and also answers this question as well as neighbor questions incorrectly in a sequence, there is a high chance that this student is disengaged while working on the particular question. However, if a student performs poorly throughout the entire lesson, DTS should not categorize the questions with abnormal response time and poor performance as disengagement since the student may be struggling with this lesson, but not just disengaged on a few questions. As noted, in this study, DTS only identifies disengaged question-answer observations when assuming that the texts and questions are within the zone of what the student can handle and the student is engaged at the beginning of each lesson. Our targeted disengaged question-answer observations are those with “abnormal” (too fast or too slow) response times, poor local performance, but adequate overall performance. Students with low performance and engagement throughout the entire lesson or study is important also. It is possible that the content of the texts or questions may be too difficult. It is important to note that these questions will not be treated as ‘disengaged’ by DTS in this study.

A more formal specification of the algorithm may lend clarity. Let $X_{i}$ be a binary random variable indicating whether the $i^{th}$ question is correctly answered (1: yes, 0: no). Overall performance is the accuracy rate that a student performs in a lesson (or up to current question *s*), defined as $\frac{{\sum^{\text{​}}}_{i=1}^{s}X_{i}}{s}$. Local performance of a question per participant in a lesson is characterized by moving averages of correctness proportion. The $k^{th}$-order moving average of $s^{th}$ question is given by $\frac{{\sum^{\text{​}}}_{i=s-k}^{s+k}X_{i}}{2k+1}$. If a student learner's overall performance in the lesson up to $s^{th}$ question is higher than a threshold $b_{u}$ and $k^{th}$-order moving average around $s^{th}$ question is below $b_{l}$, then this student is detected as ‘disengaged’. In this study, we take k=1, $b_{u}=b_{l}=0.5$ and the overall performance is calculated based on all questions in a lesson. The second part of DTS refines the filtering system by not treating well-performed question-answer observations as “disengaged” although students spent abnormal time on these questions. By additionally taking the students’ performance into consideration, DTS refines the results from the first part of DTS and largely reduces the false positives in disengagement detection confounded by other factors irrelevant to disengagement. For example, students may spend significantly more response time on a question on new or difficult material. Or a student may struggle the entire lesson and not perform well throughout the lesson (This article aims to detect specific periods where a student gets disengaged, rather than detecting disengaged students. DTS will not treat a student as disengaged when he/she is focused but struggling on this question.).

It is important to reiterate that the proposed DTS algorithm will not handle occurrences when a student is disengaged from the very beginning. These occurrences would not be counted as disengagement (even though they should be) so our predictive algorithm is conservative rather than been generous at detecting disengagement and such observations will dilute the predictive power of the DTS algorithm. It is also important to acknowledge that the algorithm has not yet been validated by self-reports of disengagement, eye tracking, and neurophysiological measures so the precise psychological status of the disengaged observations await further research. That being said, D’Mello and has colleagues (Mills et al., 2017; Faber et al., 2018; D’Mello, 2019) have proposed a decoupling algorithm of disengagement that identifies deviations between a person's self-paced reading times and projected times based on the difficulty of the material, where there is more decoupling when the times are too fast or two slow compared to the projection times; the decoupling algorithms significantly predict self-reported mind-wandering and eye tracking patterns.

### Study of Disengaged Question-Answer Observations

The proposed DTS is designed to be a real-time monitoring of disengagement in an intelligent system. Using the predicted engagement/disengagement status for individual questions, we explored the pattern of disengagement in the AutoTutor-ARC as an empirical evaluation of the algorithm (It is important to clarify that DTS was not used during the CSAL AutoTutor study. It was developed after the end of the study.). For each of 252 students, we calculated the proportions of disengaged items, including fast- and slow-disengaged question-answer observations. A k-mean clustering analysis was applied to develop student profiles on proportions of the two types of disengaged question-answer observations. K-means clustering assigns data points into groups by iteratively reassigning and re-averaging the cluster centers until the points have reached convergence (Hartigan and Wong, 1979). Grouping students with similar disengagement patterns could help us to interpret reasons for disengagement within each group of students, and use this information to guide the design of effective interventions to re-engage student users. Fang et al. (2018) performed clustering analysis on the accuracy and response time of the 252 participants and categorized the participants into four groups of adults: higher performers, conscientious readers, under-engaged readers, and struggling readers. This study compared the clusters of students with different disengagement patterns according to Fang et al.‘s four clusters. As a note, Fang et al.‘s study removed questions with extreme outliers (i.e. response time was three interquantile range higher than the third quantile).

As an independent evaluation, learning gains were analyzed on subsets of the 252 students who took three standardized tests of comprehension before and after the larger CSAL AutoTutor intervention that included AutoTutor-ARC lessons. Of the 252 participants, 205 took both pre- and post-test of the Woodcock Johnson III Passage Comprehension subtest (Woodcock et al., 2007); 143 took Reading Assessment for Prescriptive Instructional Data (RAPID) Passage Comprehension subtest develop by Lexia Learning (Foorman et al., 2017) and 142 took Reading Inventory and Scholastic Evaluation (RISE) battery developed by ETS (Sabatini et al., 2019). Fang et al. reported that the learning gains in Woodcock Johnson and RAPID tests were highest for conscientious readers, lowest for struggling readers, with higher performing readers and under-engaged readers in between (Fang et al., submitted). It has been shown that readers who invested the time to answer AutoTutor questions with a modicum of accuracy demonstrated significant learning gains on measures of comprehension (Greenberg et al., submitted), which confirms the relationship between intensity of engagement and learning. However, the analyses by Fang et al. (submitted; submitted) were conducted on the aggregate performance and response times of the lessons and items over the 4-month intervention rather focusing on engagement within a particular lesson for a particular student, the focus of the present study.

To investigate whether the learning gain is affected (presumably reduced) by disengagement, we performed paired t-test on the pre- and post-test scores of the three standardized tests after contrasting groups of students with different disengagement patterns at a fine grain level (i.e., students with a high vs. a low proportion of disengaged question-answer observations according to the DTS algorithm). These groups were obtained from the clustering analysis of the 252 participants on proportions of disengaged question-answer observations. It should be noted that these DTS-based clusters are different from the aggregate-based clusters identified by Fang et al. (submitted). We compared the two different types of clusters in this paper. Moreover, we tested the association of learning gain measured by the three standardized tests with the AutoTutor accuracy (i.e. proportions of questions correctly answered by students) of 252 participants. We first separated engaged and disengaged question-answer observations detected by the proposed DTS. For engaged (or disengaged) questions, reading comprehension at post-test was regressed onto the accuracy of engaged (or disengaged) questions adjusted by reading comprehension at pre-test.

## Results

### Accuracy for Disengaged Versus Engaged Question-Answer Observations in AutoTutor

We applied the proposed DTS algorithm to the data extracted from AutoTutor (67,235 answers to questions from 252 participants in 30 lessons). We identified 16,851 questions with “abnormal” response times, of which 3,082 were disengaged question-answer observations (including 961 fast-disengaged and 2,121 slow-disengaged question-answer observations) among the 252 participants. Table 2 presents the number of disengaged vs. engaged question-answer observations that were correctly answered. Among 3,082 disengaged question-answer observations, 569 were correctly answered, which represents 18.5% of the total disengaged question-answer observations detected by the proposed DTS algorithm. In contrast, 46,059 (71.8%) of the engaged question-answer observations were answered correctly. To test the association of the correctness and disengagement status in AutoTutor, we ran a generalized linear mixed model by letting the correctness of a question-answer observation as the response variable (1: correct, 0: incorrect) and disengagement status (1: disengaged, 0: engaged) as the predictor, and adding two random terms to adjust the correlated observations due to same student and lesson. It is shown that the odds of answering a question correctly when disengaged is only 8% of the odds when engaged. Quite clearly, when students are disengaged while working on questions in a lesson, their performance on the questions will be significantly lower than the engaged questions (Table 2, *p*-value <.001). As discussed earlier, disengagement is one of multiple reasons why students might give wrong answers to a question (e.g., the question is difficult for them, their diligent reasoning is unsuccessful), but we presume that disengagement is a very plausible explanation in a high percentage of the observations. See D’Mello (2019); Millis et al. (2017) in their validation of the decoupling model.

**TABLE 2:** Number (proportion) of correctness among disengaged vs. engaged question-answer observations.

	Number of questions correctly answered (**Correctness rate**)	Number of questions incorrectly answered (Incorrectness rate)	Total
Disengaged question-	569	2,513	3,082
answer observations	(18.5%)	(81.5%)	
Engaged question-	46,059	18,094	64,153
answer observations	(71.8%)	(28.2%)	

*Linear mixed model: coefficient = −2.56, odds ratio = exp(−2.56) = 0.08, p-value <0.001.

### Clusters of Participants and Lessons on Proportions of Disengagement

After aggregating the total number of questions from the lessons, we obtained the frequencies and proportions of disengaged question-answer observations for each of the 252 participants. The total number of questions that a student answered varied from ∼10 to ∼500, of which only a very small portion of questions (approximately 3∼9%) were disengaged question-answer observations. We identified more questions that were slow-disengaged than fast-disengaged (3.2% vs 1.4%).

Some students tend to have a higher proportion of fast-disengaged question-answer observations, whereas others have more slow-disengaged question-answer observations and yet others are high in both. To address this, a k-mean clustering analysis was performed on groups of students with similar disengagement patterns according to the DTS algorithm. Since students answered a different number of questions, we focused on the proportion (rather than the count) of fast- and slow-disengaged question-answer observations for each participant. The k-mean clustering analysis was implemented in R (version 3.6.0) on the proportions of fast- and slow-disengaged question-answer observations. We clustered the 252 participants into four groups (k = 4) according to the “elbow” method by visualizing the plot of “number of clusters” vs. “within groups sum of squares”.

**FIGURE 2:**
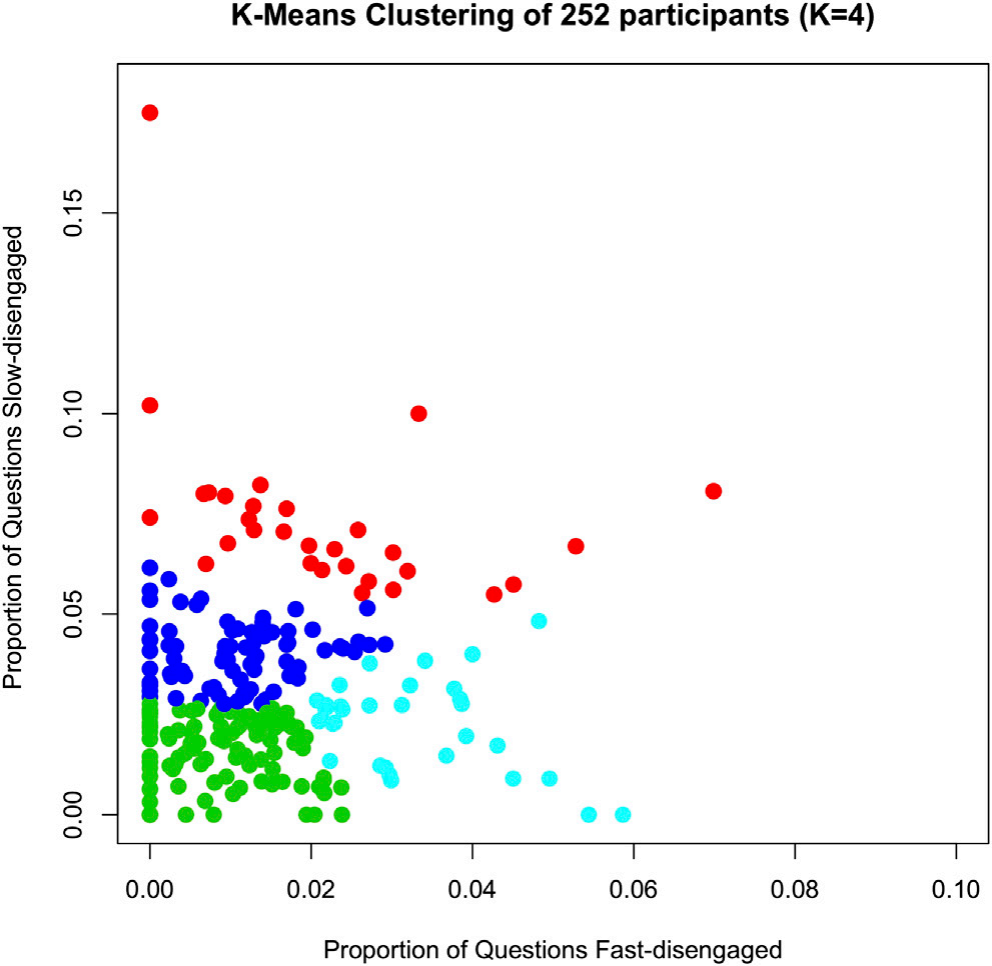
K-mean clustering of 252 participants on the proportion of fast- and slow-disengagement rate. Red dots: students with a medium-to-high proportion of fast-disengaged question-answer observations and high proportion of slow-disengaged question-answer observations (*HiFast/HiSlow*); Deep blue dots: students with a small proportion of fast-disengaged question-answer observations and medium-to-high proportion of slow-disengaged question-answer observations (*LowFast/HiSlow*); Aqua blue dots: students with a high proportion of fast-disengaged question-answer observations and small proportion of slow-disengaged question-answer observations (*HiFast/LowSlow*); Green dots: students with a small proportion of fast-disengaged question-answer observations and small proportion of slow-disengaged question-answer observations (*Engaged*).

Figure 2 plots the four clusters of participants with different disengagement patterns. The mean and standard deviation of the proportion of fast- and slow-disengaged observations in each cluster are provided in Table 3. The first cluster (red dots in Figure 2, labeled *HiFast/HiSlow* for short) represents students with a relatively medium-to-high proportion of fast-disengaged question-answer observations (2%) and a comparatively high proportion of slow-disengaged question-answer observations (7%). The second cluster (deep blue dots in Figure 2, labeled *LowFast/HiSlow* for short) includes students with a small proportion of fast-disengaged question-answer observations (1%) and a medium-to-high proportion of slow-disengaged question-answer observations (4%). The third cluster (aqua blue dots in Figure 2, labeled *HiFast/LowSlow* for short) represents students with a high proportion of fast-disengaged question-answer observations (3%) and a small proportion of medium-to-high slow-disengaged question-answer observations (2%). The last cluster (green dots in Figure 2, labeled *Engaged* for short) represents students with small proportion of fast-disengaged question-answer observations (1%) and small proportion of slow-disengaged question-answer observations (2%). Figure 2 confirms that the four clusters are visually distinct in the scatterplots. Interestingly, Figure 2 shows that there are several students with nearly zero fast-disengaged question-answer observations, but a medium-to-high proportion of slow-disengaged observations. It is possible that some of these slow-disengaged observations are not truly disengaged, but rather are instances when the student is encountering difficult questions for them. However, our assumption is that a significant percentage of the questions reflect disengagement because the performance of the students was respectable in the early phase of a lesson.

**TABLE 3:** Mean and standard deviation (SD) of fast- and slow-disengage proportions for the four clusters of participants in AutoTutor.

Cluster of disengagement from DTS	Mean (SD) of fast-Disengage rate	Mean (SD) of slow-Disengage rate
1 (Red): Disengaged- *HiFast/HiSlow*	0.02 (0.02)	0.07 (0.02)
2 (Deep Blue): Disengaged-*LowFast/HiSlow*	0.01 (0.01)	0.04 (0.01)
3 (Aqua Blue): Disengaged- *HiFast/LowSlow*	0.03 (0.01)	0.02 (0.01)
4 (Green): *Engaged*	0.01 (0.01)	0.02 (0.01)

HiFast/HiSlow—disengaged students with a medium-to-high proportion of fast-disengaged question-answer observations and high proportion of slow-disengaged question-answer observations;

LowFast/HiSlow—disengaged students with a small proportion of fast-disengaged question-answer observations and medium-to-high proportion of slow-disengaged question-answer observations;

HiFast/LowSlow—disengaged students with a high proportion of fast-disengaged question-answer observations and small proportion of slow-disengaged question-answer observations;

Engaged—students with a small proportion of fast-disengaged question-answer observations and small proportion of slow-disengaged question-answer observations.

The current classification based on local engagement (Figure 2 and Table 3) was compared with the clustering of 252 students in the Fang et al. (2018) study that classified students into four groups based on their accumulated profile over the 4-month intervention. Fang et al. (2018) categorized the 252 participants into four groups: higher performers (fast and accurate), conscientious readers (slow and accurate), under-engaged readers (fast, but lower accuracy) and struggling readers (slow and inaccurate). Table 4 compares the clusters identified in this study according to the local disengagement patterns with the ones reported in Fang et al. (2018; submitted) that considered the global performance profile. We applied chi-squared test of independence on the overlapped counts of the two sets of clusters (4-by-4 table, Table 1) and found a significant association ($\chi^{2}=26.33$, *p*-value=.002) between the clusters developed by this study and Fang et al. (2018; submitted). According to Table 4, a high percentage (52% = 50/97) of “higher performers” are classified as *Engaged* students by DTS, which is higher than “conscientious” and “struggling readers” (42% = 13/31) and much higher than “under-engaged” reader (34% = 32/93). Furthermore, when considering the students with local disengagement (including *HiFast/HiSlow*, *LowFast/HiSlow* and *HiFast/LowSlow*), the conditionalized percentages on on the slow end rather than the fast end were : higher performers (41/47 = 87%), conscientious (7/18 = 39%), struggling (14/18 = 78%), under-engaged (49/61 = 80%); low relative percentages for the conscientious readers is unexpected, but perhaps can be attributed to the relatively small number of observations.

**TABLE 4:** Comparisons of clusters of 252 participants.

Clusters according to local disengagement pattern Identified by DTS	Clusters reported in Fang et al. (2018; submitted) over 30 lessons			
	Higher performers	Conscientious readers	Struggling readers	Under-engaged reader
	Count (%)	Count (%)	Count (%)	Count (%)
1 (Red): Disengaged- *HiFast/HiSlow*	8	3	3	16
	(8%)	(10%)	(10%)	(17%)
2 (Deep Blue): Disengaged- *LowFast/HiSlow*	33	4	11	33
	(34%)	(13%)	(35%)	(35%)
3 (Aqua Blue): Disengaged- *HiFast/LowSlow*	6	11	4	12
	(18%)	(35%)	(13%)	(13%)
4 (Green): *Engaged*	50	13	13	32
	(52%)	(42%)	(42%)	(34%)
Total	97	31	31	93
(%)	(100%)	(100%)	(100%)	(100%)
Chi-squared test of independence: $\chi^{2}=26.33$, *p*-value=0.002				

HiFast/HiSlow—disengaged students with a medium-to-high proportion of fast-disengaged question-answer observations and high proportion of slow-disengaged question-answer observations;

LowFast/HiSlow—disengaged students with a small proportion of fast-disengaged question-answer observations and medium-to-high proportion of slow-disengaged question-answer observations;

HiFast/LowSlow—disengaged students with a high proportion of fast-disengaged question-answer observations and small proportion of slow-disengaged question-answer observations;

Engaged—students with a small proportion of fast-disengaged question-answer observations and small proportion of slow-disengaged question-answer observations.

The major discrepancy between the two clustering approaches can be attributed to the fact that DTS was developed to detect disengaged question-answer observations, rather than disengaged students. Thus, DTS only checks the accuracy of answers to a question locally (i.e. accuracy of neighbored questions), not globally (e.g. accuracy within lessons that accumulated over the 4-month intervention). In our study, a student is considered to be disengaged while working on a question if his/her performance on this (and neighbored) questions is lower than their global performance. If a student has a low accuracy throughout the entire lesson, DTS will count these question-answer observations as *Engaged*. In contrast, Fang et al. categorized readers with low global accuracy to “under-engaged.”

**FIGURE 3:**
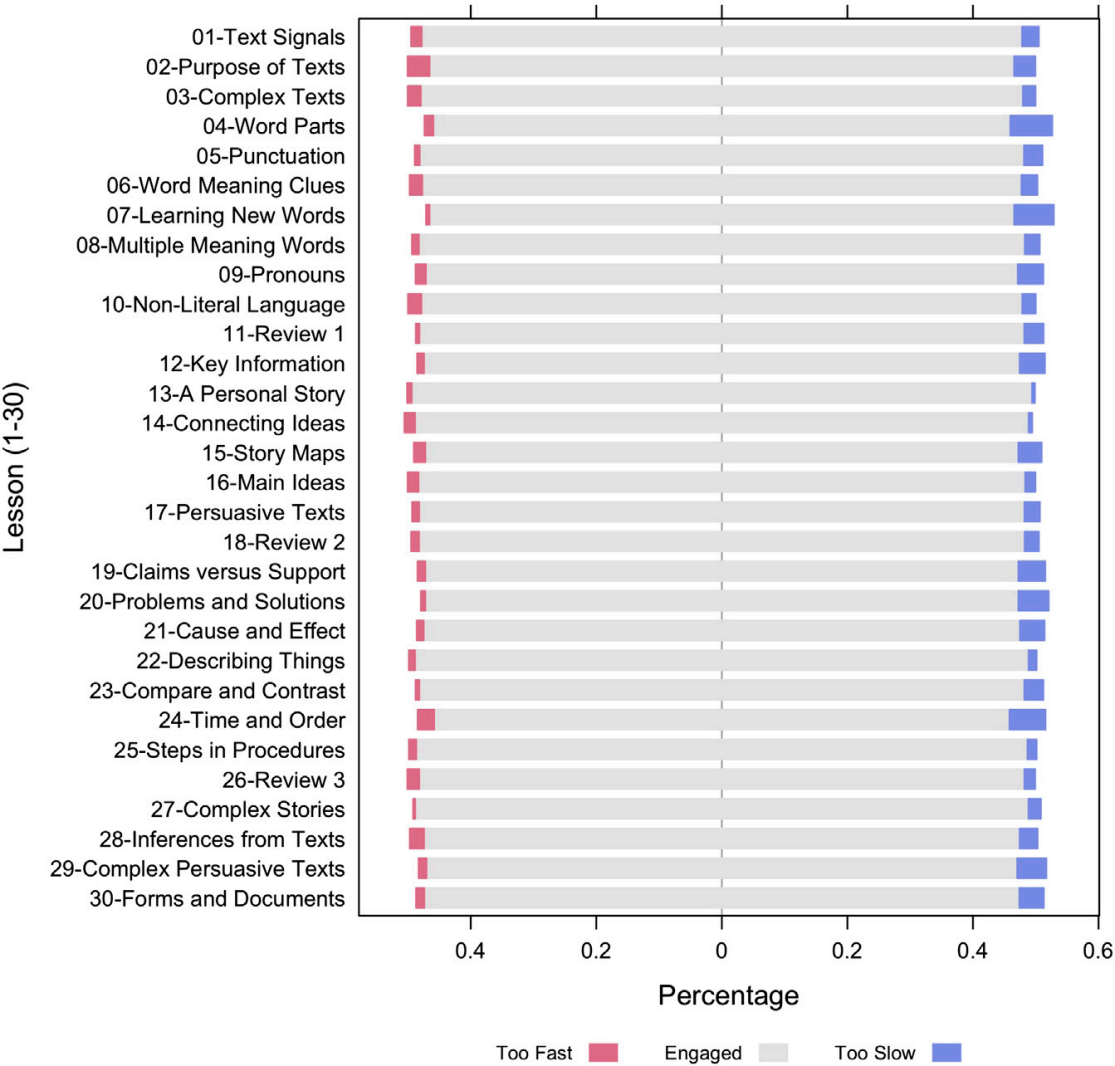
Proportions of disengaged question-answer observations from 252 participants in 30 lessons.

The next analysis computed the proportion of fast- and slow-disengaged question-answer observations among the 252 participants within each of the 30 lessons separately. Figure 3 shows these results for the 30 lessons in the approximate order that the lessons occurred in the curriculum (there were small deviations in the sequence over the course of the intervention). Figure 3 shows that the proportions of fast- and slow-disengaged observations differed among the 30 lessons. Some lessons have a larger proportion of slow-disengaged question-answer observations than others. For example, lesson #04-Word Parts and #07-Learning New Words clearly have a higher proportion of fast-disengaged question-answer observations compare to lesson #13-A Personal Story and #14-Connecting Ideas. To better understand which lessons are more (or less) likely to get students disengaged, with the fast- and slow-disengagement proportions in each lesson, we clustered the 30 lessons in terms of their disengagement pattern using k-mean clustering analysis. Exploring the disengagement pattern across lessons would provide AutoTutor designers critical information and guidance to adjust the difficulty levels of content and/or enhance the display interfaces of questions in lessons to diminish or prevent disengagement. These results are presented in Appendix A. Three groups of lessons were chosen. This first group contains lessons, such as “Text Signals”, “Purpose of Texts”, “Inferences from Texts”, has balanced proportions of fast- and slow-disengaged question-answer observations. The second group of lessons have higher proportions of slow-disengaged question-answer observations. Lessons in the second cluster include “Claims vs. Support”, “Cause and Effect”, “Persuasive Texts”, which are more advanced and difficult topics and lead to an increased slow-disengage. The proportion of both fast- and slow-disengage is low in the third group of lessons.

### Proportion of Disengaged Question-Answer Observations for Different Difficulty Levels and Theoretical Levels

**FIGURE 4:**
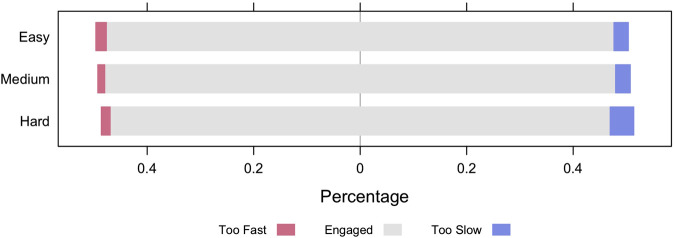
Disengagement rate (Too Fast: fast-disengaged; Too Slow: slow-disengaged) for questions of different difficulty levels.

A subset of the lessons have one or two texts with conversation-based questions woven into the lessons. Eleven of the lessons have multi-sentence texts that branched during the course of the lessons. For each of these lessons with branching texts, the AutoTutor system starts with a medium difficulty text with 8–12 questions and then branches to an easy or hard text, depending on the student's performance on the questions in the medium difficulty texts. A second set of nine lessons provide one medium level text with 10–20 questions woven into the conversation about the text. A third set of 10 lessons focused on single words or sentences rather than multi-sentence texts. These lessons had 10–30 questions that were scaled on easy, medium or difficult levels. When considering all 30 lessons, the questions at the medium difficulty level constituted the majority of questions. Since some lessons contain questions of different difficulty levels, we evaluated the proportion of fast- and slow-disengaged items stratified by difficulty levels of questions for 252 participants in the 30 lessons. Figure 4 provides the bar chart with the percentage of disengaged question-answer observations at different difficulty levels. Easy questions had a slightly larger proportion of fast-disengage compared to the other two types of questions. This can be explained by the plausible possibility that some students are bored by the easy questions and quickly click the answers. Figure 4 also indicates that the proportion of slow-disengaged observations is the highest in hard questions, which is very reasonable since students may need more time to work on hard questions; students may give up on the hard questions and get disengaged. In order to statistically assess whether the differences are reliable, we conducted a generalized linear mixed model by setting the disengagement status (1: disengaged, 0: engaged) as the response variable, level of difficulty (easy/medium/hard) as the predictor variable and adding two random terms to adjust for variability among students and lessons. The results confirmed that students tend to be disengaged more often on hard in comparison to easy questions (odds ratio = 1.5, p<.001).

The AutoTutor lessons were also scaled on four theoretical levels: Words (W), textbase (TB), Referential Situation Model (RSM), and Rhetorical Structure (RS), based on Graesser and McNamara's (2011) multilevel theoretical framework. A description of these theoretical levels is provided in Study of *Disengaged Question-Answer Observations*. For each person and lesson, we calculated the proportion of disengaged question-answer observations. To test whether the disengagement rate of lessons from one theoretical level is different from another, a linear mixed model was conducted while adjusting the correlated observations due to the same student. The results revealed that lessons in the Word (W) theoretical level had the highest disengagement rate (1% higher than RS with p=.005, 1.1% higher than TB with p=.014 and 1.7% higher than RSM with p<.001). However, the differences were surprisingly small and not different for the fast-vs. slow-disengaged items.

### Association With Learning Gains From Three Standardized Tests of Comprehension

Comprehension was evaluated by three standardized tests (Woodcock Johnson III Passage Comprehension, RISE and RAPID). There was a pretest before the 4-month intervention and a posttest at the end of it. Learning gain is calculated by the difference between pre- and post-test. To assess whether disengagement has an effect on learning gains in AutoTutor, we separated disengaged question-answer observations from the engaged ones and tested the association between learning gains from three standardized tests of comprehension and the accuracy rate (aggregated from all lessons) in AutoTutor on disengaged and engaged observations respectively. Regression analyses were conducted on the learning gains in the comprehension tests as a function of the AutoTutor intervention with engaged vs. disengaged question-answer observations. These results are presented in Table 5. Learning gains on the three standardized tests were significantly predicted by the accuracy in AutoTutor on engaged question-answer observations, but were not significant on disengaged question-answer observations. For example, when accuracy rate of engaged questions increases by one unit, the mean learning gains on Woodcock Johnson III Passage Comprehension test increase by 0.56 (*p*-value<0.001). However, the change in accuracy rate of disengaged questions is not statistically significantly associated with learning gains on Woodcock Johnson III Passage Comprehension test.

**TABLE 5:** Predicted learning gains from pretest to posttest on three standardized tests (RISE, RAPID and Woodcock-Johnson passage comprehension) from engaged vs. disengaged question-answer observations.

Types of pre- and post-tests	Engaged question-answer observations predicting learning gains (*p*-value)	Disengaged question-answer observations predicting learning gains (*p*-value)
Woodcock Johnson	0.56	−0.06
	(0.007)^a^	(0.662)
RISE	2.26	0.41
	(<0.001)^b^	(0.130)
RAPID	0.58	−0.01
	(<0.001)^b^	(0.897)

^a^ indicates that the p-value < 0.01.

^b^ indicates that the p-value < 0.001.

## Discussion and Summary

This paper provides a disengagement tracking system (DTS) with an intelligent algorithm to monitor students’ disengagement based on their response time and performance on each question during their learning process in AutoTutor. A variety of approaches have been applied to predict and track disengagement in intelligent tutoring systems (Allen et al., 2016; Bixler and D’Mello, 2013; D’Mello and Graesser, 2012). Existing disengagement/engagement detection methods mainly predict disengagement/engagement by applying supervised learning approaches using self-reported mind-wandering (Bosch and Dmello, 2019; Mills and D’Mello, 2015; Millis et al., 2017). These methods are not suitable for personalized and concurrent disengagement detection. Tracking students’ disengagement promptly would allow personalized interactions at appropriate times in order to re-engage students.

The proposed DTS consists of two steps. In the first step, the algorithm learns a student's baseline response time from his/her first 3∼5 well-performed questions in a specific lesson and creates a personalized reference of response time. This first step rests on the plausible premise that the student is engaged at the beginning of a lesson. A student is suspected to be “disengaged” on a question if the response time on a question abnormally deviates from the baseline, which is expected to be more prevalent after the initial phase of a lesson. In the second step, the algorithm checks all the 16,851 candidate disengaged question-answer observations and marks those with good overall performance in a lesson (proportion of correctly answered questions is higher than a threshold) but poor local accuracy (proportion of correctness rate in the neighbor questions but not the target question is lower than a threshold) as disengaged question-answer observations. The proposed method is derived from the time and accuracy of data in log files and does not require any self-reported reports from the participants or physiological measures of engagement. Moreover, the DTS algorithm can detect disengagement within small time spans of a minute or two rather than after a lesson or dozens of lessons have been completed. For instance, if a student is disengaged starting from the ninth question, the earliest time that the algorithm would be able to capture it is after the student completed the 11th question. The proposed algorithm offers low computational burden and can be included *in vivo* as a performance monitoring algorithm within an intelligent tutoring system.

Our study of disengaged question-answer observations in AutoTutor that were identified by DTS is consistent with the claim that disengaged observations have substantially lower accuracy on AutoTutor items whereas engaged observations high performance. This is a confirmation of the internal validity of the algorithm. Evidence of external validity was also confirmed in analyses of learning gains on comprehension skills that were measured by independent psychometric tests (Woodcock et al., 2007; Foorman et al., 2017; Sabatini et al., 2019). Learning gains on these tests were predicted by the accuracy rate of engaged question-answer observations in AutoTutor but not the disengaged observations. These two lines of evidence suggest that the evaluation and tuning of AutoTutor or other ITSs could benefit from analyzing the engagement profiles reflected in question-answer observations and that the DTS is a promising algorithm to detect disengagement.

Disengagement detection and monitoring is of course important for improving learning in conventional learning contexts as well as intelligent tutoring systems (Csikszentmihalyi, 1990; D’Mello and Graesser, 2012; Larson and Richards, 1991; Mann and Robinson, 2009; Millis et al., 2017; Pekrun et al., 2010; Pekrun and Stephens, 2012). A few ITS studies have been conducted with personalized interventions to prevent or interrupt disengaging behaviors and guide an individual learner back on track (Bosch et al., 2016; D’Mello, 2019; D’Mello and Graesser, 2012, 2012; Lane, 2015; Monkaresi et al., 2017; Woolf et al., 2010). Feedback from the proposed disengagement tracking system can elucidate factors that lead to distractions or impetuous responding. Was it the question or content difficulty or low interest in the material, poor pacing, lack of razzle dazzle, or perceived value of the learning experience? ITS can also be designed to engage the off-track student at the right time. For example, once the disengagement is identified, a conversational agent or pop-up window can express one or more of the following messages: It seems like you may be distracted. Do you need a break? Would you like to continue to learn more about XX? Alternatively, the ITS could present more difficult or easy material to optimize students’ zone of attention and learning (Graesser et al., 2016a). These interventions will hopefully encourage students to turn their attention back to the lesson. The false-positives and false-negatives generated by this DTS may or may not be problematic, depending on how DTS integrates with adaptive elements of the ITS. While this is beyond the scope of this paper, the optimal system response to disengagement may, for example, align with the optimal system response to slow engagement on difficult items. To the extent that optimal system responses overlap, DTS errors are not problematic. In cases where the appropriate system response should differ, these offer opportunities to improve DTS.

There are a number of limitations in this study that call for follow-up research. First, we assumed that the log-transformed response time follows a normal distribution, and hence an “abnormal” response time can be identified if a log-transformed response time falls outside of $z^{\text{*}}$ standard deviation of its mean. The resulting distributions of the log-transformed response times confirmed that the distributions were normal. However, some data sets might not exhibit a normal distribution. To accommodate any severely skewed or heavy-tailed distributions, the proposed method can be revised by replacing the mean and standard deviation with more robust alternatives, e.g. median and median absolute deviation (MAD) as suggested by (Miller, 1991; Leys et al., 2013). Thus, a student will be suspected to be disengaged on a question if the response time on this question is below or above three MAD from the median response time of engaged items. These possibilities can be explored in future research.

Second, the DTS algorithm assumes that questions in a lesson are similar/interchangeable in terms of the lesson content and difficulty. Figure 3 and Appendix A display the variations among the 30 lessons. Somewhat surprisingly, there were very small and primarily nonsignificant differences when comparing the theoretical levels of the lessons (words, textbase, situation model, rhetorical structure). Our study revealed that the proportion of slow-disengaged observations is higher in the comparatively hard questions (see Figure 4). As discussed earlier, the literature has confirmed that disengagement and mindwandering increase with the difficulty of expository reading materials (D’Mello, 2019; Feng et al., 2013; Miller, 1991; Mills et al., 2017). In our future studies, we may improve the DTS by adding a factor that annotates text/item difficulty or difficulty transitions to prevent falsely discovering slow-disengagement when the materials given to a student branches to harder materials.

Third, the algorithm does not detect situations when the student is disengaged from the material at the beginning of the lesson. For the DTS to be meaningfully applied to AutoTutor, we assume that texts/questions given to students are suitable for them and have some modicum of value and/or interest. This is a plausible initial assumption because the lessons focus on subject matters that have value for struggling adult readers (e.g., comprehending a rental agreement or a job form) or are interesting to adults. Hence, students presumably start out engaged in most of the questions and may be disengaged on a number of questions some time later. If a student is disengaged in the beginning of a lesson or disengaged from most of the questions, DTS would need to be adjusted with a different algorithm to improve its predictions.

Fourth, there are a number of other situations that the DTS algorithm would need to be modified to handle. The proposed algorithm does not consider any intervention to re-engage the students. DTS needs to be adjusted if any intervention action is taken after a disengaged question is detected. If users encounter frequent technical issues in the early/testing stage of a new ITS system, the data should take that into consideration. DTS run the risk if identifying “false alarms in disengagement” or “misses in disengagement observations” if the questions at the early phase of a lesson are unusual and fail to calibrate their performance when engaged.

In summary, DTS provides an algorithm that can automatically predict/monitor disengaged behaviors in other learning environments that collect self-paced responses to question-answer items during training. It was designed for, but not limited to, the AutoTutor-ARC system. It can be tailored to fit any ITSs. In the proposed algorithm, only the response time and accuracy of each question are utilized to predict disengagement since they are the only relevant items that are recorded by AutoTutor. If other predictors or measurements, such as item difficulty, self-reported engagement or student's gaze patterns captured by a commercial eye tracker are available in different intelligent tutoring systems, they can be incorporated into the proposed DTS with appropriate modifications to the proposed algorithm. These other sources of data can also be used to validate the DTS algorithm. Of course, these other measures may be difficult or impossible to collect when scaling up a learning system in the real world.

## Data Availability

The datasets presented in this study can be found in online repositories. The names of the repository/repositories and accession number(s) can be found below: LearnSphere, https://datashop.memphis.edu/ProjectPermissions?id=76.
