# Supplementary material for: Automated Disengagement Tracking Within an Intelligent Tutoring System
Source: Front Artif Intell. 2021 Jan 20;3:595627. doi: 10.3389/frai.2020.595627 (PMC7971516; doi:10.3389/frai.2020.595627)
Supplement: Supplementary file 1 [file table1.pdf]

APPENDIX A

Table S1. Clusters of lessons with different disengagement patterns

Lesson Name	Fast-disengage (%)	Slow-disengage (%)
**Cluster 1: Proportion of fast- and slow-disengaged question-answer observations are balanced and at medium level**		
01-Text Signals	1.7	2.7
02- Purpose of Texts	3.5	3.4
03-Complex Texts	2.1	2.0
06-Word Meaning Clues	2.0	2.5
10-Non-Literal Language	2.1	2.1
15-Story Maps	1.8	3.7
28-Inferences from Texts	2.3	2.9
**Cluster 2: Proportion of fast-disengaged question-answer observations is low and proportion of slow-disengaged question-answer observations is high**		
04-Word Parts	1.4	6.7
07-Learning New Words	0.6	6.3
09-Pronouns	1.6	4.1
12- Key Information	1.1	4.0
19-Claims versus Support	1.3	4.3
20-Problems and Solutions	0.7	4.8
21-Cause and Effect	1.1	3.9
24-Time and Order	2.6	5.7
29-Complex Persuasive Texts	1.3	4.6
30-Forms and Documents	1.3	3.9
**Cluster 3: Proportion of fast-disengaged question-answer observations is low and proportion of slow-disengaged question-answer observations is at low-medium level.**		
05-Punctuation	0.8	3.0
08-Multiple Meaning Words	1.1	2.4
11-Review 1	0.6	3.1
13-A Personal Story	0.7	0.5
14-Connecting Ideas	1.7	0.6
16-Main Ideas	1.7	1.6
17-Persuasive Texts	1.1	2.5
18-Review 2	1.3	2.3
22-Describing Things	1.0	1.3
23-Compare and Contrast	0.6	3.0
25-Steps in Procedures	1.2	1.5
26-Review 3	1.9	1.7
27-Complex Stories	0.3	2.0
